# Rolling Bearing Fault Diagnosis Model Based on Multi-Scale Depthwise Separable Convolutional Neural Network Integrated with Spatial Attention Mechanism

**DOI:** 10.3390/s25134064

**Published:** 2025-06-30

**Authors:** Zhixin Jin, Xudong Hu, Hongli Wang, Shengyu Guan, Kaiman Liu, Zhiwen Fang, Hongwei Wang, Xuesong Wang, Lijie Wang, Qun Zhang

**Affiliations:** 1 Coal Mine Intelligent Equipment Research Center of Shanxi Province, Taiyuan University of Technology, Taiyuan 030024, China; jinzhixin@tyut.edu.cn (Z.J.); 19143331920@163.com (X.H.); gsytyut@163.com (S.G.); liukaiman2000@163.com (K.L.); fangzhiwen1209@link.tyut.edu.cn (Z.F.); wanghongwei01@tyut.edu.cn (H.W.); 2 School of Safety and Emergency Management Engineering, Taiyuan University of Technology, Taiyuan 030024, China; 3 School of Mining Engineering, Taiyuan University of Technology, Taiyuan 030024, China; 4 State Key Laboratory of Intelligent Mining Equipment Technology, Taiyuan 030032, China; wxs-119@163.com (X.W.); tzznck123@126.com (L.W.); zhangqun6@163.com (Q.Z.); 5 Xinjiang Intelligent Equipment Research Institute, Aksu 843000, China

**Keywords:** rolling bearing, fault diagnosis, Gramian angular difference field, multi-scale depthwise separable convolutional neural network, transfer learning

## Abstract

In response to the challenges posed by complex and variable operating conditions of rolling bearings and the limited availability of labeled data, both of which hinder the effective extraction of key fault features and reduce diagnostic accuracy, this study introduces a model that combines a spatial attention (SA) mechanism with a multi-scale depthwise separable convolution module. The proposed approach first employs the Gramian angular difference field (GADF) to convert raw signals. This conversion maps the temporal characteristics of the signal into an image format that intrinsically preserves both temporal dynamics and phase relationships. Subsequently, the model architecture incorporates a spatial attention mechanism and a multi-scale depthwise separable convolutional module. Guided by the attention mechanism to concentrate on discriminative feature regions and to suppress noise, the convolutional component efficiently extracts hierarchical features in parallel through the multi-scale receptive fields. Furthermore, the trained model serves as a pre-trained network and is transferred to novel variable-condition environments to enhance diagnostic accuracy in few-shot scenarios. The effectiveness of the proposed model was evaluated using bearing datasets and field-collected industrial data. Experimental results confirm that the proposed model offers outstanding fault recognition performance and generalization capability across diverse working conditions, small-sample scenarios, and real industrial environments.

## 1. Introduction

Rolling bearings play an essential role in mechanical systems, as their operating state is closely related to the efficiency and stability of mechanical systems [1]. When a bearing malfunctions, it can result in unplanned equipment downtime, considerable financial loss, and potential threats to human safety [2]. As such, exploring techniques for bearing fault detection is crucial for enhancing the operational safety and cost-effectiveness in industrial systems.

Compared with traditional methods, deep learning techniques demonstrate superior capabilities in feature extraction, representation learning, and information processing [3]. Moreover, the robust generalization capacity and adaptability of these models have driven the widespread adoption of deep learning in bearing fault detection research [4].

Pan et al. [5] introduced a fault diagnosis framework in which raw vibration signals are directly fed into a convolutional neural network (CNN) for feature extraction, followed by a long short-term memory (LSTM) network for sequential modeling and fault classification. Zhang et al. [6] utilized the short-time Fourier transform (STFT) to transform temporal vibration signals into joint time–frequency representations, which are then input to a deep neural architecture incorporating hierarchical normalization to enhance fault identification accuracy. Li et al. [7] processed the raw signals through wavelet transform, combined a CNN with a Kolmogorov–Arnold network (KAN) for feature extraction, and effectively improved the diagnostic accuracy. He et al. [8] proposed an adaptive graph framelet convolutional network that dynamically constructs graph structures from vibration signals and employs framelet-based graph convolution for robust fault diagnosis in high-speed train bogie bearings under intense noise and variable load conditions. Wang et al. [9] proposed a multi-level information fusion network that integrates deep and empirical features via a hybrid attention mechanism (combining self-attention and mutual attention) to mitigate diagnostic conflicts in bearing fault diagnosis. Li et al. [10] proposed a fault diagnosis method for rolling bearings that utilizes contrastive learning with a novel spectrum-based loss function. This approach enables accurate fault detection using only normal state signals via a multi-branch deep residual shrinkage network architecture.

However, in practice, fault-related features in vibration signals are often distributed across multiple scales. Most conventional approaches rely on single-scale feature extraction, which limits their ability to fully characterize complex and heterogeneous fault patterns. To address this limitation, recent studies have explored the design of multi-scale convolutional neural networks to extract and integrate features from different receptive fields. Chao et al. [11] proposed a novel multi-scale cascaded structure capable of capturing multi-resolution features embedded in raw data, while leveraging residual connections to mitigate performance degradation in deeper networks. Li et al. [12] enhanced the representational capacity of their model by applying random sampling to preprocess the input signal and utilizing multiple convolutional kernels of varying sizes for feature extraction. He et al. [13] applied one-dimensional convolutional layers for initial feature learning, and subsequently employed dilated convolutions with four different dilation rates to capture features across multiple temporal resolutions.

Although multi-scale convolutional neural networks have improved the representation of fault characteristics, many of these models simply concatenate features extracted from different scales without explicitly modeling their relative importance. More critically, some branches may introduce noise or redundant information, which can mislead the classification process and degrade diagnostic accuracy [14]. Therefore, there remains a need for more refined architectures that can selectively integrate multi-scale features while suppressing irrelevant or misleading signals.

In practical applications, the effectiveness of diagnostic models is highly dependent on the availability of ample annotated datasets for supervised learning, which is often difficult to achieve in engineering practice [15]. Additionally, since variations in operating conditions can significantly affect the vibration characteristics of bearings, many existing methods struggle to effectively handle these changes, leading to a decline in fault identification accuracy [16].

To overcome the aforementioned difficulty, employing transfer learning (TL) techniques for fault detection has attracted significant interest in recent studies [17]. Liu et al. [18] introduced an innovative adaptation approach utilizing a numerical simulation-guided one-dimensional cyclic generative adversarial network (GAN) that transforms simulation signals into near-real signals for model training. This approach effectively preserves fault information, making the generated signals realistic. Huang et al. [19] addressed the challenge posed by substantial discrepancies within the source dataset by utilizing the maximum mean discrepancy (MMD) metric to identify suitable samples, thereby constructing a refined source dataset that facilitates effective model learning. Han et al. [20] introduced a hierarchical transfer learning framework designed to proficiently adjust to the conditional distribution of unlabeled target samples by aligning feature distributions across source and target domains, thereby reducing the discrepancy with the source domain representations. Li et al. [21] developed an advanced adversarial transfer framework designed to tackle the challenge of diagnosing novel faults within the target environment by utilizing established fault information originating from the source dataset. While these approaches partially mitigate the domain shift between the source and target datasets, they remain constrained in delivering robust and comprehensive solutions, particularly under varying operational scenarios and scarce target domain samples.

To tackle this challenge, the study introduces a rolling bearing fault diagnosis model based on a multi-scale depthwise separable convolutional neural network integrated with a spatial attention mechanism (SA-MSDSCNN). The key contributions and novel aspects of this research are outlined below:

(1) This work proposes a novel framework that first transforms vibration signals into images using the Gramian angular difference field (GADF), enriching temporal structure representation. A multi-scale depthwise separable convolutional module is then introduced to simultaneously model feature correlations in both channel and spatial dimensions, enabling efficient and precise extraction of discriminative fault patterns at multiple resolutions.
(2) To focus the model on diagnostically critical information, a spatial attention mechanism is incorporated. This mechanism dynamically weights and fuses the multi-scale features, significantly amplifying the response of discriminative characteristics while suppressing the influence of redundant and interfering information, thereby enhancing diagnostic robustness under complex operating conditions.
(3) To address challenges of scarce training samples and dynamic operational condition distributions, we adopt a transfer learning strategy. Optimized model parameters from a source domain are transferred and fine-tuned using limited target domain data, enabling rapid adaptation to new conditions and significantly improving fault diagnosis performance and generalization capability in data-scarce scenarios. Comprehensive validation demonstrates the method’s superior accuracy, stability, and generalization across diverse scenarios.

## 2. Related Theories and Methods

### 2.1. Gram Angle Difference Field

The Gramian angular difference field (GADF) is a nonlinear mapping technique that converts univariate temporal sequences into visual representations in a two-dimensional space [22]. Its core idea is to construct a structured feature space through polar coordinate mapping and angle difference computation, thereby enhancing the representation of dynamic patterns in time series data. The generation of GADF involves the following three key steps:

Step 1: Normalization of Sequential Time Data

The original time series is first normalized to eliminate dimensional inconsistencies and constrain its value range. A min–max normalization method is adopted, defined as:

(1) $${\hat{x}}_{i}=\frac{\left(x_{i}-\max(X))+(x_{i}-\min(X)\right)}{\max\left(X\right)-\min\left(X\right)},\;{\hat{x}}_{i}\in \left[-1,1\right]$$

where *X* is the original time series and ${\hat{x}}_{i}$ represents the normalized value.

Step 2: Polar Coordinate Mapping

The normalized one-dimensional time series is then projected into a polar coordinate system. Specifically, the time series is transformed as follows:

(2) $$\left\{\begin{array}{l} \theta_{i}=\arccos({\hat{x}}_{i}),\;-1\leq {\hat{x}}_{i}\leq 1,\;{\hat{x}}_{i}\in \hat{X}\\ r_{i}=\frac{t_{i}}{N},\;t_{i}\in N \end{array}\right.$$

where $\theta_{i}$ represents the angle corresponding to the normalized value ${\hat{x}}_{i}$ in polar coordinates, $t_{i}$ indicates the timestamp at that position, *N* serves as a fixed coefficient for adjusting the radial extent of the polar coordinate framework, and *r_i_* is the radius derived from the timestamp.

Step 3: Construction of GADF

Finally, the GADF matrix is generated based on the angle differences in the polar coordinate space by Equation (3).

*G_i_*_,*j*_ = [sin(*θ_i_* − *θ_j_*)] (3)

### 2.2. Multi-Scale Depthwise Separable Convolution Module

**Figure 1:**
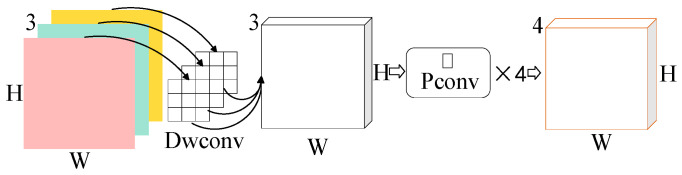
Depthwise separable convolution.

Depthwise separable convolution decomposes conventional convolution into two separate processes: spatial filtering (depthwise convolution) followed by channel-wise combination (pointwise convolution). In the depthwise stage, each input channel is convolved independently using its own kernel, thereby facilitating spatial feature extraction per channel [23]. Subsequently, the pointwise convolution—a 1 × 1 convolution—performs a linear combination of the depthwise outputs, integrating information across channels. This architecture decouples spatial and channel-wise feature modeling, offering improved computational efficiency and representation capability compared to conventional convolution, which simultaneously processes both dimensions [24]. The structure of depthwise separable convolution is illustrated in Figure 1.

In contrast to standard convolutions with fixed kernel sizes, multi-scale convolution leverages kernels of varying sizes to extract features at different receptive fields. The multi-scale convolution captures more comprehensive information across multiple scales [25].

**Figure 2:**
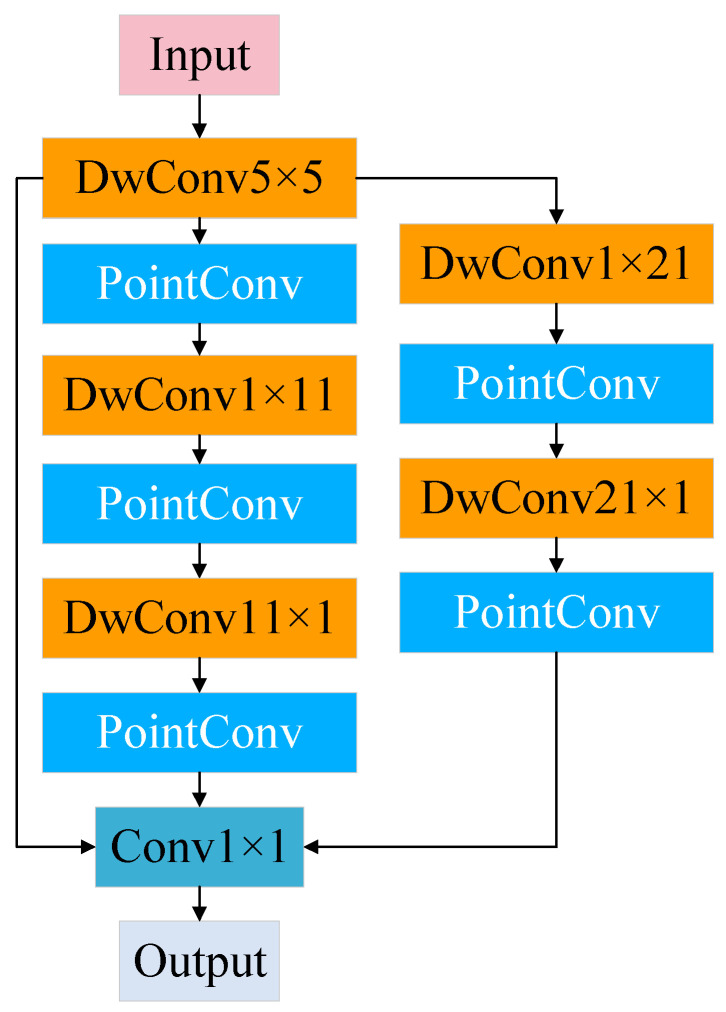
Multiscale depthwise separable convolution module.

To strengthen the model’s capacity for recognizing features at multiple resolution levels, a multi-scale depthwise separable convolution module is proposed. The architecture of it is shown in Figure 2. This module integrates multi-scale convolution with the depthwise separable convolution mechanism. Specifically, it performs parallel convolutional operations with kernels of various sizes, enabling the extraction of both local and long-range dependencies from different spatial perspectives. Each branch applies a depthwise convolution, followed by a pointwise convolution to fuse channel-wise features.

To reduce the number of output channels and merge features from all multi-scale branches, a 1 × 1 convolution is introduced at the end of the module. This operation aggregates the outputs from all branches into a unified feature map.

Additionally, the module adopts a strip convolution strategy by decomposing standard 2D convolutional kernels into two sequential 1D convolutions. This architecture simultaneously reduces the model parameters and computational complexity, while enhancing the ability to capture directional features without sacrificing receptive field size.

By combining the strengths of multi-scale convolution, depthwise separable convolution, and strip convolution, the proposed module enables efficient modeling of spatial structures and channel interactions. The resulting discriminative feature representation is particularly advantageous for fault diagnosis tasks.

### 2.3. Spatial Attention Module

To enhance the model’s ability to focus on key regions, we introduce an improved spatial attention module within the feature extraction network. This module builds upon the convolutional block attention module (CBAM) [26] with targeted optimizations. The module first constructs spatial attention maps by aggregating average pooling and max pooling operations on the spatial dimension of the input feature map.

(4) $$F_{avg}=\mathrm{AvgPool}\left(F\right)$$

(5) $$F_{\max}=\mathrm{MaxPool}\left(F\right)$$

The pooled feature maps are then concatenated along the channel dimension.

(6) $$F_{\mathrm{contact}}=\left[F_{\mathrm{avg}};\;F_{\max}\right]$$

In this paper, the original standard convolution in CBAM is replaced with a more efficient depthwise separable convolution to further improve the local sensitivity of feature extraction. First, a depthwise convolution is applied to capture spatial dimensional correlations:

(7) $$F=\mathrm{DConv}\left(F_{\mathrm{contact}},\;\mathrm{size}=7\right)$$

Then, a pointwise convolution is performed to integrate inter-channel features. Finally, Sigmoid is applied to scale the convolution result between 0 and 1, generating the spatial attention weights.

(8) $$M_{S}\left(F\right)=\sigma \left(PC\mathrm{onv}(F)\right)$$

**Figure 3:**
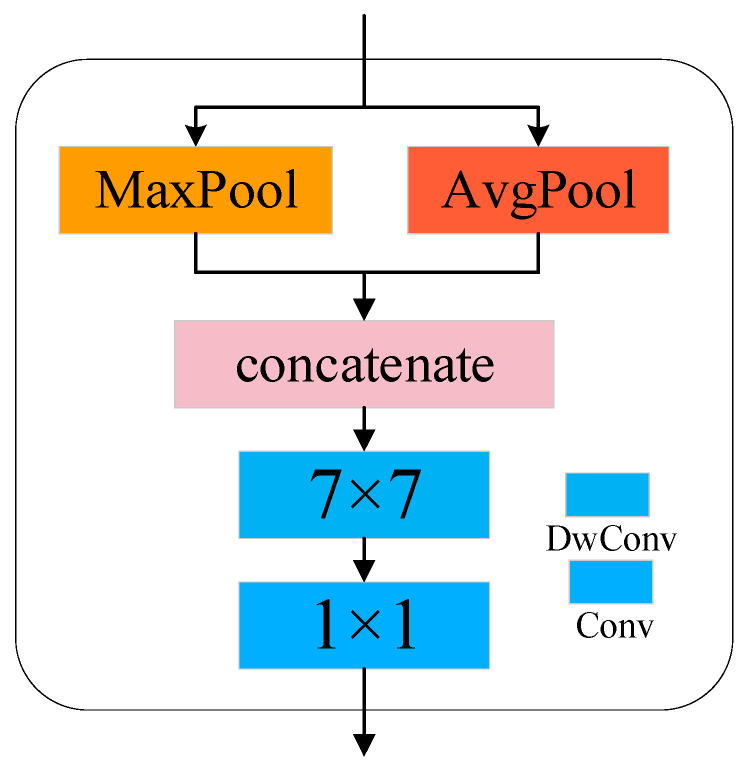
Spatial attention module.

The configuration of the spatial attention module is illustrated in Figure 3.

The spatial attention module dynamically focuses on task-relevant key regions by generating a spatial weight map, enhancing the model’s perception of spatial structures. Simultaneously, it suppresses interference noise from irrelevant background areas through dynamic adjustment of weight distribution across spatial positions.

## 3. Fault Diagnosis Model and Diagnosis Process

### 3.1. Fault Diagnosis Model and Diagnosis Process Under Constant Operating Conditions

**Figure 4:**
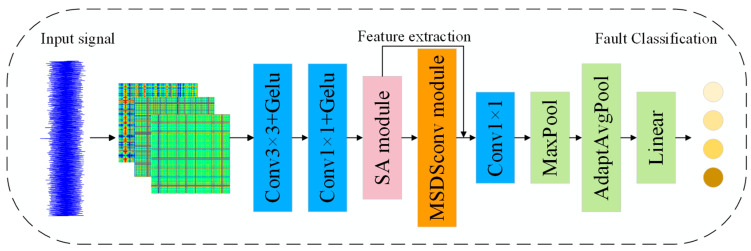
The architecture of SA-MSDSCNN.

The architecture and workflow of the model are depicted in Figure 4. To efficiently extract potential fault features in vibration signals, this paper first employs the Gramian angular difference field (GADF) method to encode raw vibration signals.

In the backbone of the feature extraction network, several key modules are sequentially integrated to progressively extract and fuse multi-scale discriminative features. Initially, a set of standard convolutional layers with kernel sizes of 3 × 3 and 1 × 1, activated by the Gaussian error linear unit (GELU) function, is used for primary feature modeling of the GADF images. GELU achieves smooth nonlinearity through Gaussian probabilistic weighted activation and adaptively regulates negative values without manual parameter tuning, and its smoothness ensures gentler gradient changes during backpropagation, effectively mitigating gradient vanishing issues [27].

Subsequently, a spatial attention module is introduced to enhance the model’s responsiveness to key regions. Following this, the core feature extraction is performed by the proposed multi-scale depthwise separable convolution module. This module integrates depthwise separable convolution paths with various receptive field sizes and incorporates a strip convolution structure to effectively capture both local and long-range features at different scales, enabling separate modeling of spatial and channel dimensions.

The spatial attention mechanism is applied once again to reinforce discriminative features and suppress redundant ones. A 1 × 1 convolution layer is used for channel integration, followed by max pooling and adaptive average pooling operations to compress the spatial dimensions while preserving global information. Finally, the fault classification is performed through the softmax function, and cross-entropy loss is applied to quantify prediction errors.

### 3.2. Fault Diagnosis Process Under Varying Operating Conditions

#### 3.2.1. Model-Driven Domain Adaptation

Model-based transfer learning is a method of transfer learning that focuses on directly leveraging model structures or parameters trained on a source domain. By fine-tuning the model parameters using data from the target domain, knowledge from the source task is transferred to the target task, thereby improving the performance on the target task [28]. This approach is particularly effective when the target data are limited. In fault diagnosis, transfer learning using data under different operating conditions can significantly enhance model performance under limited sample scenarios.

#### 3.2.2. Few-Shot Fault Diagnosis Process Under Variable Working Conditions

**Figure 5:**
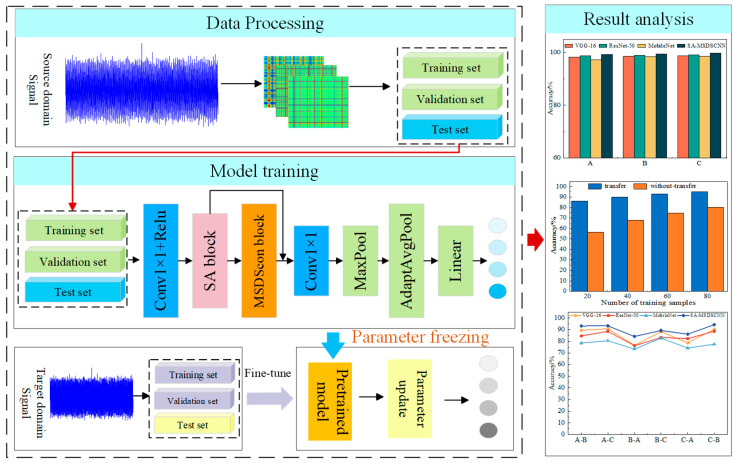
Fault diagnosis framework under varying operating conditions.

In this paper, data from different working conditions are utilized to implement transfer learning. After pre-training on the source dataset, the learned parameters are preserved. Subsequently, the model is fine-tuned on all layers using data from the target domain. Figure 5 illustrates the complete fault diagnosis workflow incorporating this transfer learning strategy.

In order to achieve effective fault diagnosis with limited samples across diverse operating scenarios, this study employs a transfer learning framework. The comprehensive procedure for model training and knowledge transfer is outlined as follows:

Step 1: Data Preprocessing

Vibration signals of rolling bearings are collected under multiple working conditions. The one-dimensional time series is then encoded into two-dimensional images using the GADF method, enhancing the temporal structure and pattern representation capabilities. The processed data are subsequently divided into training, validation, and test sets to support model training and evaluation.

Step 2: Source Domain Model Training

A comprehensive dataset from a chosen operating condition serves as the source domain to form the training set, which is subsequently fed into the SA-MSDSCNN model for training. Once the training reaches the specified number of epochs, the resulting model parameters are stored as the pre-trained weights to facilitate later transfer learning.

Step 3: Few-Shot Transfer and Fine-Tuning in the Target Domain

A limited subset of samples from the target domain is utilized to form the training dataset. The weights of the model pre-trained on the source domain are employed for initialization, followed by fine-tuning of all network layers to progressively align with the statistical properties of the target domain. The training process proceeds until the predetermined iteration count is met or the validation metrics converge.

Step 4: Model Testing and Result Output

Test samples from the target domain are fed into the fine-tuned model to generate classification outputs. The diagnostic effectiveness is then assessed to validate the model’s generalization ability and precision in scenarios characterized by limited data and varying operating conditions.

## 4. Experimental Results and Analysis

The model is executed under the Windows 11 operating system, with an NVIDIA RTX-3080Ti. The programming language used is Python 3.11, and the framework is Pytorch. The reported results are the averaged metrics derived from 10 separate experimental trials.

### 4.1. Bearing Dataset

**Figure 6:**
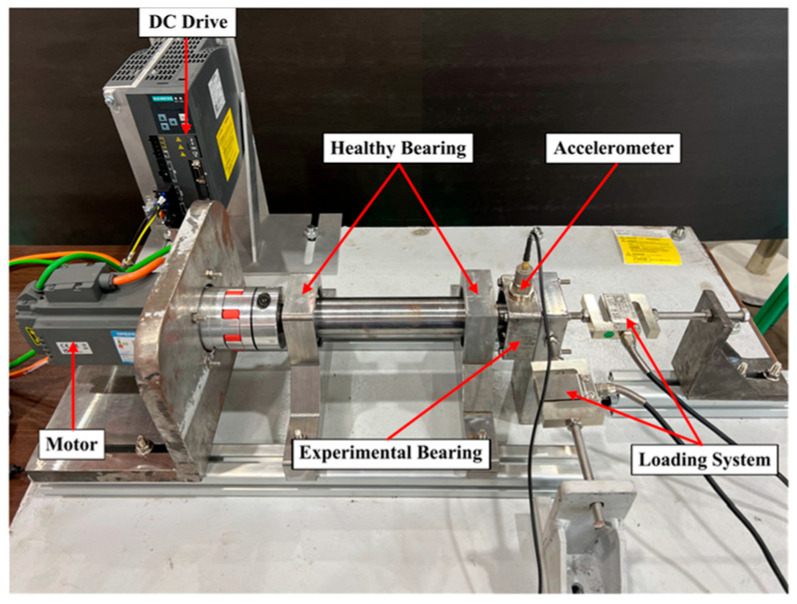
Experimental platform.

In this study, the bearing fault dataset collected from a simply supported beam with a shaft-end loading fault simulation platform is used as the experimental data [29]. The experimental setup is illustrated in Figure 6. The tested bearing is an angular contact ball bearing (model: 7205B). Inner race, outer race, and rolling element faults were simulated using electric spark erosion. For the inner race and outer race faults, both were simulated with a width of 0.3 mm and a depth of 0.5 mm. The rolling element fault was simulated with a diameter of 0.6 mm and a depth of 0.5 mm. Three working conditions were set: 600 r/min–1.5 KN, 1200 r/min–1 KN, and 1800 r/min–0.5 KN. Datasets collected under these varying conditions are denoted as A, B, and C, respectively.

During the experiment, vibration signals under different fault conditions were sampled using a sliding window with a size of 1024 points. Each 1024-point data segment constituted a sample and was transformed into a 224 × 224 image. For each working condition, 320 samples per fault type were collected, resulting in 1280 samples per condition. Detailed experimental dataset specifications are provided in Table 1.

### 4.2. Experimental Parameter Tuning

**Figure 7:**
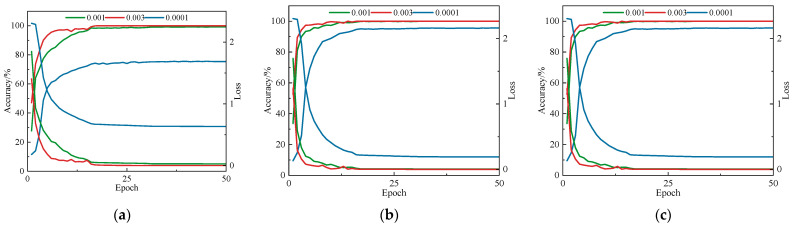
Model training accuracy and loss curves across varying learning rate settings: (**a**) Dataset A; (**b**) Dataset B; (**c**) Dataset C.

Under the condition of keeping other hyperparameters unchanged, the batch size was set to 16, and three different learning rates—0.0001, 0.001, and 0.003—were tested for experiments on different datasets. The results are shown in Figure 7.

Among the three datasets, the configuration utilizing a learning rate of 0.003 exhibited enhanced convergence efficiency and improved classification precision. It achieved high accuracy and low loss within fewer epochs, demonstrating strong training efficiency and generalization capability. Consequently, the learning rate was fixed at 0.003 for model training.

The selection of convolution kernel size exerts a substantial influence on the model’s feature representation capability during the extraction process [30]. To strengthen the extraction of hierarchical feature representations, this paper introduces a multi-scale depthwise separable convolution module and conducts systematic comparative experiments on the fault diagnosis performance using different kernel combinations under the three working conditions.

With the network structure and other hyperparameters fixed, the convolution kernels within the module were set to [3,5], [3,7], [7,11], and [11,21]. Each condition-specific dataset was partitioned into training, validation, and test subsets at a 7:2:1 ratio. The corresponding results are presented in Table 2.

Experimental results demonstrate that the proposed [11,21] kernel combination achieved optimal performance across all operating conditions, attaining accuracies of 99.3%, 99.4%, and 99.7% on the three datasets, respectively. With an average accuracy of 99.3%, this configuration significantly outperformed alternative kernel combinations.

### 4.3. Experimental Results and Performance Analysis

#### 4.3.1. Fault Diagnosis Under Constant Working Conditions

**Figure 8:**
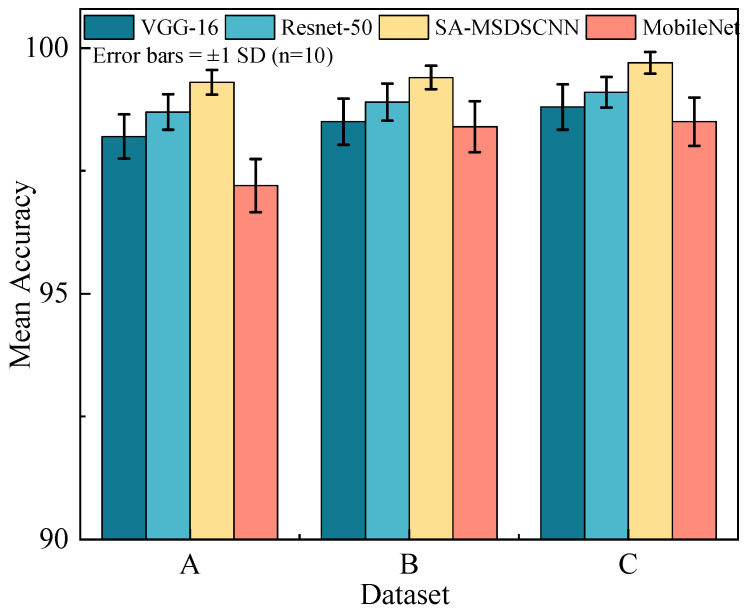
Experimental results of different models under different datasets.

To systematically analyze the proposed model’s performance in fault identification across multiple operating environments, experiments were conducted under three constant speed and load settings. Each condition-specific dataset was partitioned into training, validation, and test subsets at a 7:2:1 ratio. Using identical data partitions and experimental configurations, mainstream convolutional neural networks—VGG16, MobileNet, and ResNet-50—were selected as baselines for comparative analysis. Fault diagnosis results across all conditions are presented in Figure 8.

Experimental results confirm that the proposed model achieved higher diagnostic accuracy and exhibited superior robustness compared to baseline models across all operating conditions, as evidenced by its consistently lower standard deviation in performance across datasets in Figure 8. The architecture integrates multi-scale receptive fields, enabling effective extraction of both localized features and long-range contextual patterns across spatial scales. Furthermore, the implementation of axis-aligned strip convolution enhances directional feature extraction while preserving receptive field dimensions. The incorporated spatial attention mechanism dynamically weights feature importance, focusing computational resources on regions of high diagnostic significance. These design elements collectively enable robust generalization performance and consistent results under varying conditions, demonstrating strong applicability for fault detection in diverse operational environments.

#### 4.3.2. Few-Shot Fault Diagnosis Under Variable Conditions

To evaluate the generalization capability of the proposed model for few-shot fault diagnosis across varying operating conditions, cross-domain transfer learning experiments were conducted. In each experiment, one operating condition was designated as the source domain and another as the target domain.

The model pre-trained on source domain data was transferred as an initialized network. This initialized model was then fine-tuned on the target domain. For fine-tuning, training sets of 20, 40, 60, and 80 samples were randomly selected while maintaining a fixed test set of 320 samples. Full parameter fine-tuning was performed to adapt the model to target domain characteristics. The experimental results are presented in Table 3.

Experimental results show that as the number of training samples in the target domain increased, the diagnostic accuracy under the target condition improved significantly, verifying the effectiveness of the transfer learning strategy in few-shot scenarios. Notably, when the number of training samples reached 80, the average accuracy achieved 96.65%, approaching the performance of fully supervised training. Even in the extreme case of only 20 training samples, the proposed model still maintained an average accuracy of 84.50%, demonstrating its strong transferability and generalization capability.

**Figure 9:**
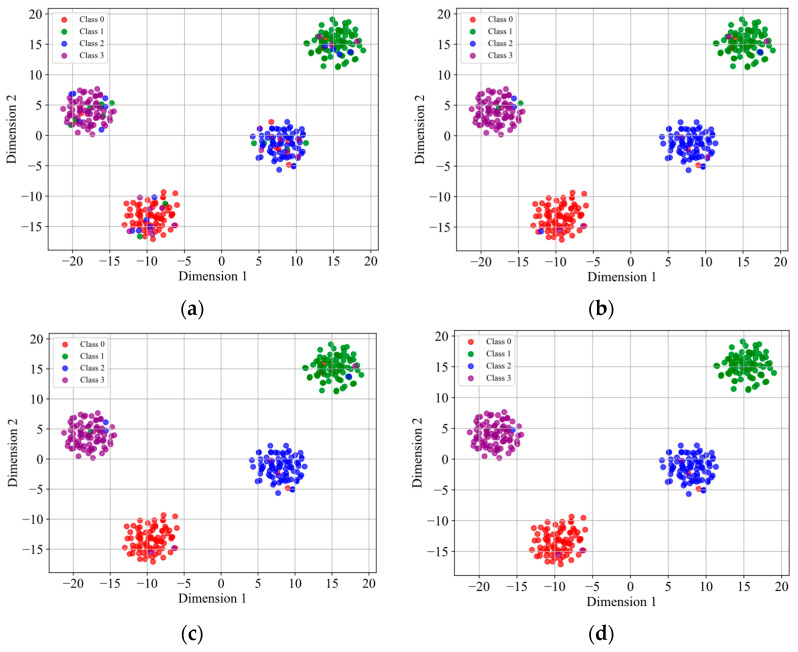
Transfer learning diagnosis: t-SNE projection of model outputs (source: A, target: B): (**a**) 20 samples; (**b**) 40 samples; (**c**) 60 samples; (**d**) 80 samples.

To visualize classification performance across sample sizes, t-distributed Stochastic Neighbor Embedding (t-SNE) was applied to the model’s output features. Using dataset A as the source domain and dataset B as the target domain, Figure 9 presents feature visualizations from a representative transfer learning experiment.

The t-SNE visualization in Figure 9 demonstrates that the model preserved discriminative power across varying sample quantities, with classification accuracy improving progressively as sample size increased. This trend aligns with the quantitative accuracy improvements, confirming consistent behavior between feature space organization and final diagnostic outcomes.

**Figure 10:**
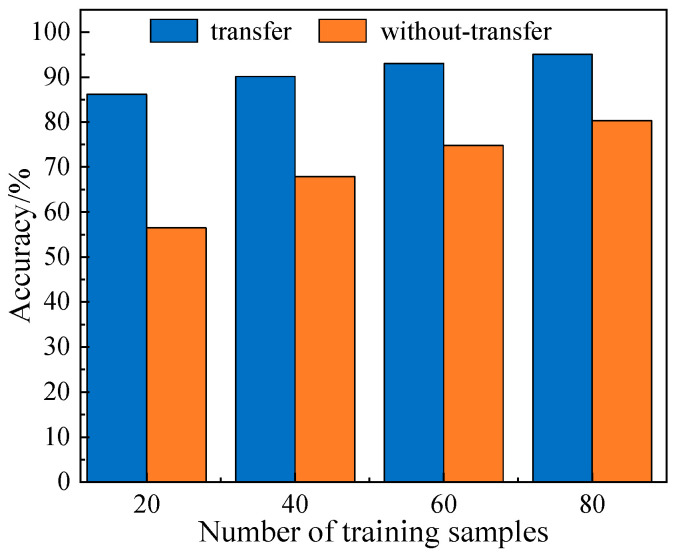
Comparison of results with and without transfer learning.

To further validate the effectiveness of transfer learning under few-shot conditions, a control experiment was conducted. Fault diagnosis was performed by inputting target domain data into both an uninitialized network and a model pre-trained on the source domain. The transfer learning diagnostic results represent average accuracy rates across all source-target domain pairs at varying training sample sizes. The performance differences under varying training sample sizes were compared, and the results are shown in Figure 10.

As shown in Figure 10, the model employing transfer learning consistently achieved significantly higher accuracy across all training sizes compared to the model without transfer learning. This comparison clearly demonstrates that by transferring knowledge from the source domain, the model can rapidly acquire superior feature representation in the target domain, thereby improving the accuracy and robustness of diagnosis in few-shot scenarios.

**Figure 11:**
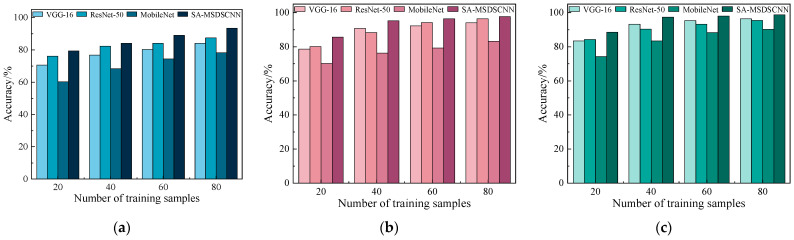
Comparison of classification accuracy across models with varying sample sizes: (**a**) dataset A; (**b**) dataset B; (**c**) dataset C.

To comprehensively evaluate the diagnostic efficacy of the proposed model under different conditions, additional comparative experiments were conducted under the same settings. The mean diagnostic values were calculated by using the other two datasets as source domains for the same target domain. The models mentioned earlier were used for benchmarking, and their classification accuracy under different target-domain training sample sizes was compared. The results are shown in Figure 11.

The results indicate that the proposed model consistently outperformed the baseline models across all sample sizes. In particular, with 20 and 40 training samples, the model showed a more pronounced advantage, reflecting its strong feature representation ability and generalization performance.

Moreover, as the number of training samples increased, all models exhibited performance gains. However, SA-MSDSCNN consistently maintained the leading position, validating the effectiveness of its architectural design in multi-scale feature extraction and cross-domain feature adaptation.

#### 4.3.3. Different Input Representations

**Figure 12:**
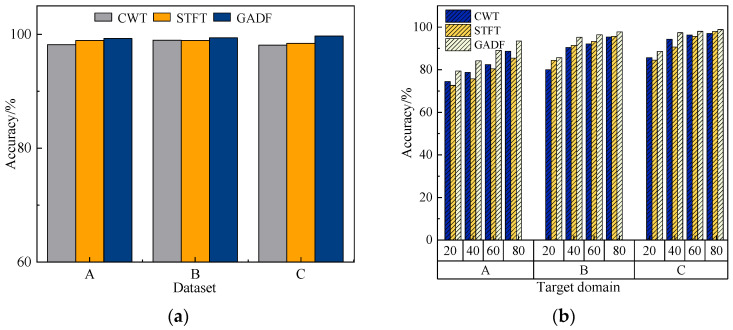
Comparison of results across different inputs: (**a**) constant operating conditions; (**b**) variable operating conditions.

To validate the effectiveness of the time–frequency representation method based on the Gramian angular difference field (GADF) in fault diagnosis tasks, two commonly used time–frequency transformation methods—continuous wavelet transform (CWT) and short-time Fourier transform (STFT)—were selected as baselines for comparison. Experiments were conducted under both constant and variable working conditions while keeping the rest of the model architecture unchanged, for the purpose of analyzing the effects of diverse time–frequency transforms on the model’s predictive capability. The diagnostic results correspond to the mean values obtained when the other two datasets serve as source domains for the same target domain. The experimental results are shown in Figure 12.

As illustrated in the figure, under constant working conditions, all three methods are capable of extracting meaningful features for classification. However, GADF consistently achieved higher accuracy across all three datasets compared to CWT and STFT, demonstrating its superior representational capability within the proposed model. This led to improved diagnostic performance in both few-shot and cross-condition scenarios, thereby confirming the rationality and superiority of the chosen time–frequency representation approach.

#### 4.3.4. Robustness Analysis

**Figure 13:**
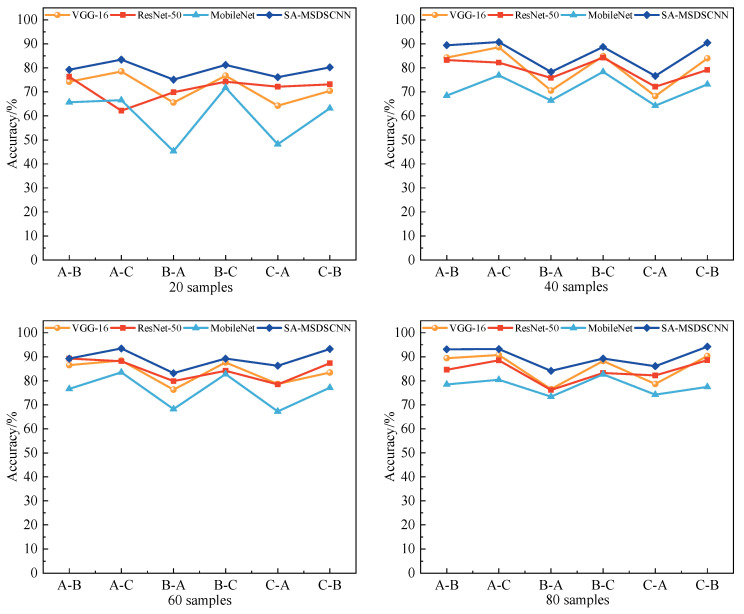
Diagnostic performance comparison across methodologies under 10 dB Gaussian noise conditions.

**Figure 14:**
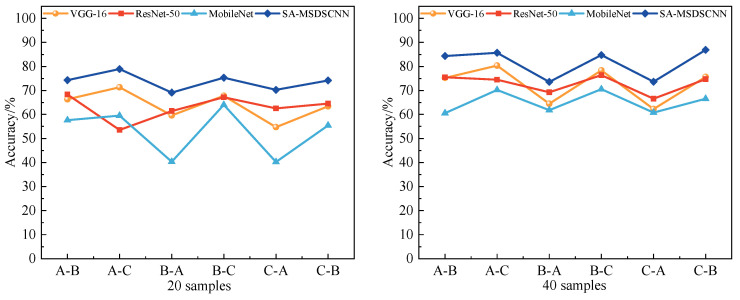

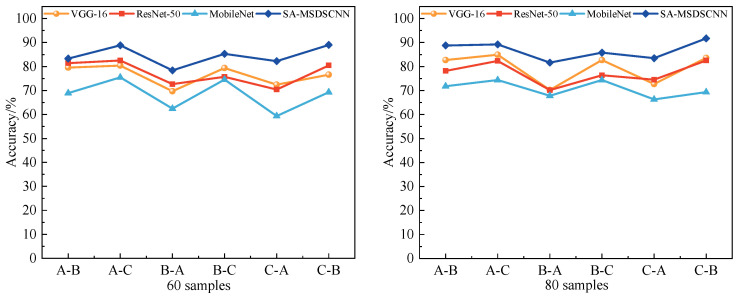
Diagnostic performance comparison across methodologies under 2 dB Gaussian noise conditions.

To further evaluate the model’s robustness in practical environments, Gaussian noise at signal-to-noise ratio (SNR) levels of 10 dB and 2 dB was added to the test dataset. Comparative analyses were performed across varying sample sizes and different source-to-target domain transfer settings. The performance of VGG-16, ResNet-50, MobileNet, and the proposed SA-MSDSCNN model was evaluated under these noisy conditions, and the results are depicted in Figure 13 and Figure 14.

The results indicate that although all models experienced a decline in diagnostic accuracy due to noise interference, the proposed model consistently maintained the highest accuracy across all transfer scenarios and varying sample sizes, highlighting its superior robustness. As illustrated in Figure 13 and Figure 14, the proposed model achieved notably better diagnostic performance under both 10 dB and 2 dB Gaussian noise conditions. Notably, under the highly challenging 2 dB condition with 20 samples in all transfer scenarios, the proposed model achieved an average accuracy of 73.67%, significantly outperforming the best baseline 63.86% by 9.81%. Furthermore, when noise increased from 10 dB to 2 dB, the accuracy drop for the proposed model was markedly smaller than that of the best baseline, demonstrating its enhanced resistance to degradation under severe noise. This strong performance can be attributed, in part, to the integration of the spatial attention (SA) block, which enhances the model’s capability to emphasize salient and noise-invariant regions of the input feature maps. By adaptively suppressing irrelevant or noisy information while amplifying diagnostically significant features, the SA block effectively strengthens the model’s resistance to external disturbances. These findings demonstrate that the proposed model not only possesses excellent transferability and few-shot learning capabilities, but also excels in robust fault feature extraction under noisy environments, which contributes to its effectiveness in operational industrial fault identification.

#### 4.3.5. Ablation Experiment

**Figure 15:**
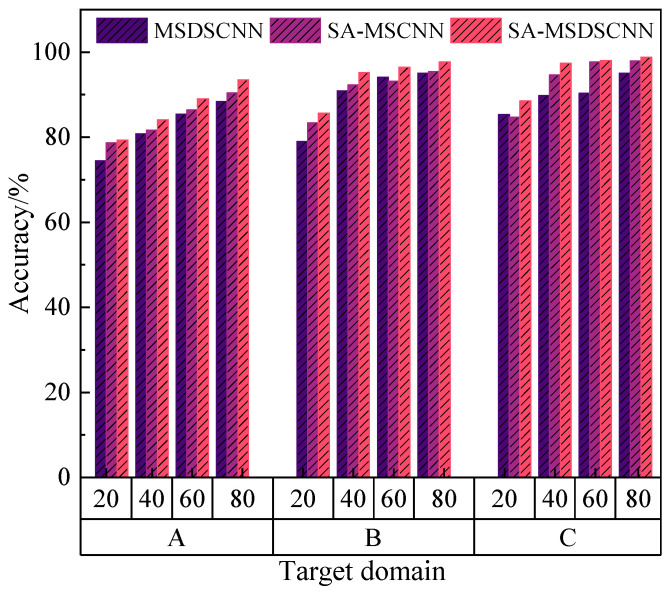
Ablation experiment.

For analyzing the role of separate components in the overall model performance, an ablation study was conducted by evaluating three model variants: (1) MSDSCNN—removing the attention module; (2) SA-MSCNN—replacing depthwise separable convolution with standard convolution while retaining the attention mechanism; and (3) SA-MSDSCNN—the complete proposed model. Experiments were carried out on three datasets, and the classification accuracy of each model was compared under varying training sample sizes. These results correspond to the mean diagnostic accuracy achieved when each of the remaining two datasets served as the source domain for the same target domain. The results are shown in Figure 15.

The results demonstrate that the SA-MSDSCNN achieved the most favorable outcome across all datasets and sample sizes, significantly outperforming its ablated versions. This confirms that the synergistic integration of multi-scale design, attention mechanisms, and depthwise separable convolutions considerably enhanced the model’s classification performance. Specifically, the inclusion of the attention module improved discrimination of essential feature regions by the model compared to MSDSCNN. Meanwhile, replacing standard convolution with depthwise separable convolution in SA-MSCNN notably reduced model complexity and parameter count while maintaining high performance, thereby enhancing generalization.

The ablation study thus validates the structural rationality and effectiveness of the proposed SA-MSDSCNN, highlighting that each module plays a critical role in boosting diagnostic accuracy.

### 4.4. Engineering Validation

To reinforce the evidence supporting the model’s applicability in real-world scenarios, an engineering test was conducted in the underground coal mining environment at the E2312 fully mechanized coal face of Gaohe Energy, Shanxi. A wireless vibration sensor was installed on the bearing end of the motor output shaft of the scraper conveyor to collect real-time vibration signals.

The dataset consists of vibration signals collected from February to November 2023 during normal operation of the coal face. It includes signals from the motor output-end bearing in normal conditions, as well as those corresponding to three typical fault types: inner ring fault, outer ring fault, and rolling element fault. After preprocessing, 240 samples were extracted for each fault. Every sample contains 1024 data points. Training and testing samples were assigned according to an 8:2 distribution.

**Figure 16:**
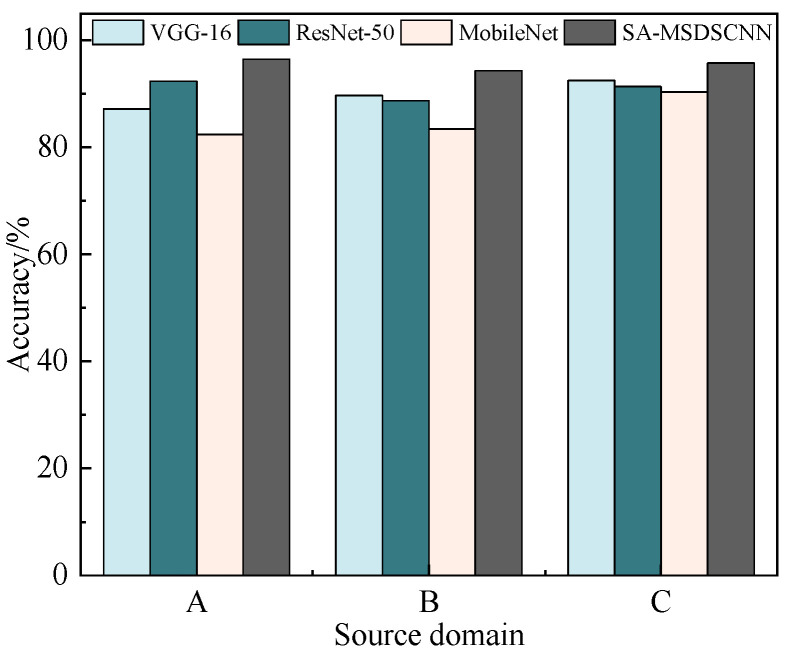
Accuracy comparison across pre-trained models.

The collected signals were encoded using the Gramian angular difference field (GADF) method and then fed into the proposed model for fault diagnosis. The model utilized pre-trained weights derived from different source domains. The experimental results are illustrated in Figure 16.

These results demonstrate the model’s capacity to effectively diagnose real-world bearing faults under complex underground industrial conditions, further confirming its robust generalization ability and practical engineering value.

**Figure 17:**
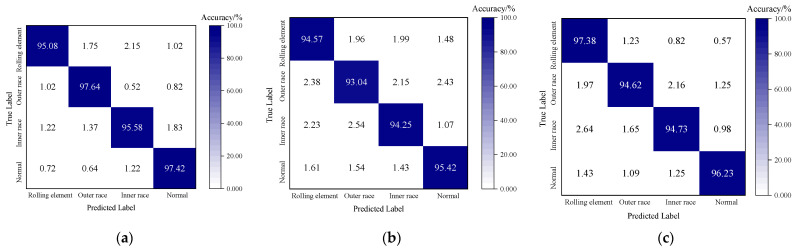
Confusion matrix: (**a**) dataset A; (**b**) dataset B; (**c**) dataset C.

To comprehensively visualize the classification performance of the SA-MSDSCNN model, a confusion matrix is presented in Figure 17. The results demonstrate a high degree of consistency between the predicted labels and the true labels. These findings indicate that the proposed algorithm exhibited excellent fault classification capability when applied to real-world data, further validating its strong potential for practical deployment in industrial applications.

## 5. Conclusions

This paper proposes SA-MSDSCNN, a fault diagnosis method for rolling bearings. The framework first converts vibration signals via Gramian angular difference field (GADF) transformation, enhancing the spatial representation of temporal sequences. Compared to alternative transformations (continuous wavelet transform—CWT and short-time Fourier transform—STFT), GADF better preserves temporal dynamics, thereby strengthening fault pattern discriminability. In terms of model architecture, SA-MSDSCNN combines spatial attention mechanisms and multi-scale depthwise separable convolutions to explicitly model and reweight information along the spatial and channel dimensions. This enhances the model’s responsiveness to key feature regions and effectively captures fault characteristics at multiple scales.

Under constant working conditions, the model attained a mean accuracy rate in fault diagnosis of 99.4% across multiple datasets, significantly outperforming the baseline models. In cross-condition few-shot transfer learning scenarios, the model achieved average accuracies of 84.50%, 92.23%, 93.47%, and 96.65% for the training samples with 20, 40, 60, and 80 training samples, respectively, demonstrating exceptional adaptability under data scarcity. Furthermore, on a real-world industrial bearing vibration dataset, SA-MSDSCNN still achieved an average fault recognition rate of 95.50%, fully illustrating its strong robustness and generalization ability in complex practical environments.

SA-MSDSCNN shows significant advantages in fault information extraction, adaptation to multiple working conditions, and few-shot transfer learning, indicating promising potential for engineering applications.

## Figures and Tables

**Table 1:** Experimental data.

Dataset	Fault Type	Class Label	Number of Samples	Input Shape
A/B/C	Normal	0	320	224 × 224
	Inner race	1	320	224 × 224
	Outer race	2	320	224 × 224
	Rolling element	3	320	224 × 224

**Table 2:** Testing accuracy for different convolution kernel sizes.

Kernel Sizes	A	B	C	Average Accuracy
[3,5]	96.3	98.8	99.1	97.5
[3,7]	98.1	98.8	99.4	98.2
[7,11]	97.2	98.5	99.6	98.4
[11,21]	99.3	99.4	99.7	99.4

**Table 3:** Classification accuracy of models under varying operating conditions.

Source Domain	Target Domain	Training Sample			
		20	40	60	80
A	B	85.91	95.94	96.46	98.14
	C	91.63	97.83	98.15	99.12
B	A	83.33	84.72	87.85	93.44
	C	85.42	96.93	97.85	98.46
C	A	75.36	83.47	90.16	93.45
	B	85.37	94.49	96.37	97.29
Average accuracy		84.50	92.23	93.47	96.65

## Data Availability

The original contributions presented in this study are included in the article. Further inquiries can be directed to the corresponding author.
